# Increased serum NfL and GFAP levels indicate different subtypes of neurologic immune‐related adverse events during treatment with immune checkpoint inhibitors

**DOI:** 10.1002/ijc.35328

**Published:** 2025-01-20

**Authors:** Christina Schmitt, Katharina J. Müller, Steffen Tiedt, Nora Kramer, Isabel Manger, Samuel Knauss, Leonie Müller‐Jensen, Petra Huehnchen, Wolfgang Boehmerle, Florian Schöberl, Lucie Heinzerling, Louisa von Baumgarten

**Affiliations:** ^1^ Department of Dermatology and Allergy LMU University Hospital Munich Germany; ^2^ Department of Neurology LMU University Hospital Munich Germany; ^3^ Institute for Dementia and Stroke Research, LMU University Hospital Munich Germany; ^4^ Department of Dermatology and Allergy University Hospital Erlangen, Friedrich‐Alexander‐University Erlangen‐Nürnberg (FAU) Erlangen Germany; ^5^ Department of Neurology with Experimental Neurology Charité—Universitätsmedizin Berlin, corporate member of Freie Universität Berlin and Humboldt‐Universität zu Berlin Berlin Germany; ^6^ Berlin Institute of Health (BIH) at Charité—Universitätsmedizin Berlin Berlin Germany; ^7^ Berlin Institute of Health at Charité—Universitätsmedizin Berlin, BIH Charité Junior Clinician Scientist Program Berlin Germany; ^8^ Department of Neurosurgery LMU University Hospital Munich Germany; ^9^ German Cancer Consortium (DKTK), Partner Site Munich, a Partnership Between DKFZ and LMU University Hospital Munich Germany; ^10^ Bavarian Cancer Research Center (BZKF) Munich Germany

**Keywords:** biomarker, immune checkpoint inhibitors, melanoma, neurological immune‐related adverse events, neurotoxicity

## Abstract

Neurologic immune‐related adverse events (nirAEs) represent rare, yet severe side effects associated with immune checkpoint inhibitor (ICI) therapy. Given the absence of established diagnostic biomarkers for nirAEs, we aimed to evaluate the diagnostic utility of serum Neurofilament Light Chain (NfL) and Glial Fibrillary Acidic Protein (GFAP). Fifty‐three patients were included at three comprehensive cancer centers, of these 20 patients with manifest nirAEs and 11 patients with irHypophysitis. Controls included patients without any irAE (*n* = 8) and other irAEs (*n* = 14). Using a single‐molecule enzyme‐linked immunosorbent assay (Simoa), serum levels were measured prior to, during and after the manifestation of (n)irAEs in 80 samples. Symptom severity of the (n)irAEs was graded according to the Common Criteria for Adverse Events (CTCAE) version 5.0. Serum NfL levels were significantly higher in the nirAE group (*n* = 20) compared to irHypophysitis (*n* = 11; *p* = .0025) and controls (*n* = 22; *p* = .0384). Subgroup analysis demonstrated a significant elevation of NfL in nirAEs of the peripheral nerves (PNirAE) in contrast to neuromuscular syndromes (NMirAE) (*p* = .0260). GFAP levels were highest in patients with nirAE affecting the central nervous system (CNSirAE) compared to PNirAE and NMirAE (*p* = .0064). Symptom severity of nirAEs was associated with increased levels of NfL and GFAP (*p* = .0069, .0092). Individuals with elevated NfL levels exhibited less favorable outcomes of the (n)irAEs (*p* = .0199). Measurement of NfL and GFAP may be helpful for the differentiation of the broad spectrum of nirAEs and may serve as an indicator of symptom severity. Further investigation is needed to evaluate their potential as diagnostic and prognostic biomarkers.

## Graphical abstract

What's new?

Neurological immune‐related adverse events from immune checkpoint inhibitor therapy are rare, but potentially life‐threatening. Here, the authors identified serum neurofilament light chain (NfL) and GFAP, an astrocyte activation marker, as promising biomarkers. Serum NfL levels were significantly increased in patients with neurological immune‐related adverse events compared to controls. NfL could distinguish neurological immune‐related adverse events affecting the central nervous system and peripheral nerves and those exclusively involving the neuromuscular junction and/or muscles. Moreover, GFAP distinguished central nervous system from other immune‐related adverse events. NfL levels in neurological immune‐related adverse events correlated with the severity and outcomes of neurological side effects.

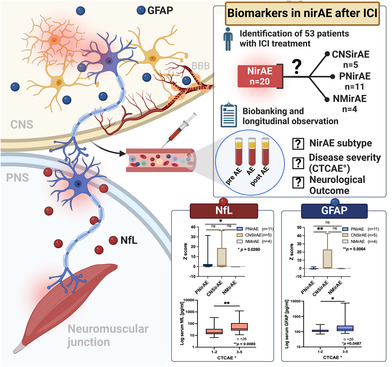

## 1 INTRODUCTION

While immune checkpoint inhibitors (ICI) demonstrate remarkable clinical efficacy, their usage can lead to immune‐related adverse events (irAEs), which may persistently and substantially reduce the quality of life of cancer patients.^1^, ^2^ Neurologic immune‐related adverse events (nirAEs) are comparatively rare with incidence rates of 1%–5% and present with variable clinical manifestations in the central (i.e., myelitis, meningitis, encephalitis) as well as the peripheral nervous system (i.e., neuropathies, myasthenic syndromes, myositis).^3^, ^4^ Severe nirAEs have been reported in up to 2.8% of all patients receiving ICI therapy,^4^ potentially leading to long‐term disability or even death. Management of nirAEs includes high dose steroids in the first line and intravenous immunoglobulins (IVIGs) as well as immunosuppressive drugs in second line.^5^, ^6^ The pathogenesis of nirAEs remains incompletely understood, but is thought to involve pro‐inflammatory cytokines, excessive activation and proliferation of autoreactive T cells, a decrease of *T*_reg_ functions as well as direct antigen effects (i.e., for cases with irHypophysitis due to binding to CTLA4).^4^, ^7^ NirAEs typically occur within the first 6 months after initiation of ICI treatment with an increased risk observed for combination therapy involving anti‐CTLA4 and anti‐PD1 inhibitors. Particularly for patients suffering from encephalitis and myasthenia‐myositis overlap syndromes, mortality rates of up to 20%–30% have been reported.^4^, ^8^, ^9^ However, there are presently no reliable biomarkers available to identify and differentiate nirAEs as well as to predict and measure their onset or severity.^3^, ^4^ Various predictive biomarkers exist for non‐neurological irAEs, such as Ki‐67+ CD8+ T cells in peripheral blood, mononuclear cells, IL‐6, and CRP, none however usable in clinical routine care or with a lack of specifity as CRP. Yet, the available data on potential biomarkers for nirAEs is limited.^10^, ^11^, ^12^ Recently, however an association with effector memory type 1 and CD8+ T cells and central memory T cells was reported. NirAEs showed no association with preceding neurotropic infections.^8^, ^13^ In, general, nirAEs were shown to be correlated with a higher cerebrospinal fluid cell count. However, it has to be considered, that nirAEs affecting the peripheral nervous system structures probably will not follow this correlation.^14^

Biomarkers for nirAEs should predict and assess acute and long‐term nervous system injuries in individual patients. They must exhibit high specificity to the nervous system and align with different hypothesized injury mechanisms. We therefore selected the combination of Glial Fibrillary Acidic Protein (GFAP) and Neurofilament Light Chain (NfL) as candidate diagnostic markers that meet these criteria. NfL is a structural intermediate filament within axons and has been well established as a diagnostic and also promising prognostic biomarker of neuroaxonal destruction in various neurological diseases affecting the central nervous system (CNS) as known for multiple sclerosis, and peripheral nervous system (PNS) as neuropathies.^15^, ^16^, ^17^ Regarding neurotoxicity, serum NfL levels were shown to correlate with the risk and the severity of neurologic side effects (i.e., Immune‐Effector‐Cell‐associated‐Neurotoxicity‐Syndrome, ICANS) after chimeric antigen receptor T (CART) cell therapy.^18^, ^19^ Recently, an early elevation of NfL after ICI treatment was described in a single case with irEncephalomyelitis.^20^ GFAP, a cytoskeletal protein predominantly expressed in astrocytes, reflects astrocyte activation and thus was shown to be increased in various CNS‐pathologies (i.e., traumatic brain injury, encephalitis, NMOSD),^21^ as well as in immune related neurotoxicity after CART treatment.^22^ Preclinical data demonstrated an upregulation of either GFAP alone or the combination of GFAP and NfL in murine multiple sclerosis models, associated with active states of inflammation and the disease peak.^23^, ^24^

In our study, we explored the combined use of NfL and GFAP as candidate biomarkers to detect and differentiate nirAEs and their severity in patients undergoing ICI treatment.

## 2 METHODS

### 2.1 Patient inclusion

**FIGURE 1:**
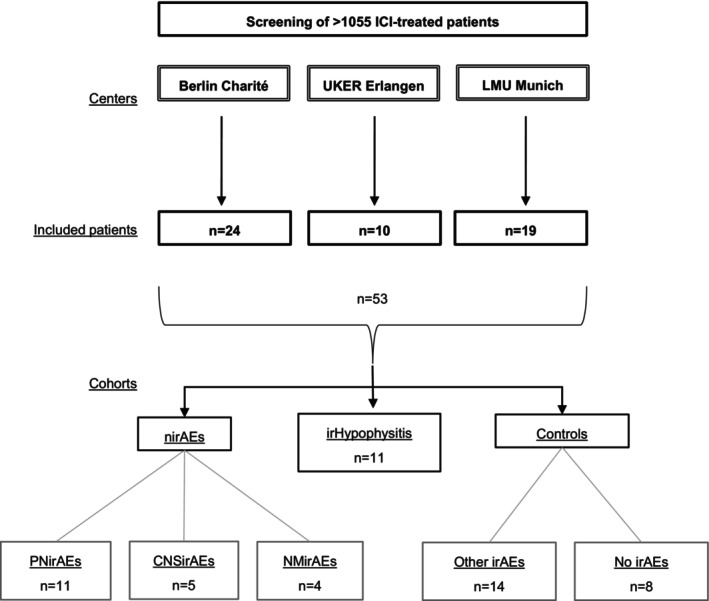
Patient flow. Utilizing the MelAutim database, we identified patients with nirAEs across three centers in Germany: Berlin Charité, University Hospital Erlangen (UKER), and the University Hospital of Munich, LMU. More than 1000 patients were screened. Based on the predominant neurologic syndrome of the nirAEs, patients were categorized into three subgroups: Peripheral nerve/nerve root affection, PNirAE (*n* = 11), affection of the central nervous system, CNSirAE (*n* = 5), or affection of the neuromuscular junction and/or muscles, NMirAEs (*n* = 4). Cases with irHypophysitis (*n* = 11) were analyzed separately. Control cohorts consisted of patients with other irAEs (*n* = 14) or no irAEs (*n* = 8).

In our multicenter clinical study, conducted within the framework of “MelAutim—Profiling of Melanoma Patients and Patients with Autoimmunity”, patients with nirAEs were identified retrospectively across three German centers: Berlin Charité, University Hospital Erlangen (UKER), and the University Hospital of the LMU Munich. MelAutim, funded by the Bundesministerium für Bildung und Forschung (BMBF), investigates autoimmunity in the context of immunotherapy. This study focused on cancer patients undergoing ICI therapy, with and without irAEs. None of the patients received chemotherapy, concomitantly. In collaboration with neurological departments at the three university hospitals, we screened more than 1000 patients experiencing irAEs. Based on the predominant neurological syndrome, patients with nirAEs were categorized into three subgroups: those involving the central nervous system (CNSirAE), peripheral nerves and nerve roots (PNirAE), or neuromuscular adverse events (NMirAEs) (Figure 1). Control cohorts included patients with other or no irAEs. IrHypophysitis cases were considered a distinct cohort to prevent any over‐ or underreporting bias for the nirAE subgroup. To date, the classification of irHypophysitis as partly a nirAE or an isolated endocrine irAE is still debated.^25^, ^26^, ^27^, ^28^ Given that patients with irHypophysitis commonly exhibit pituitary gland enlargement, inclusion in the control group was considered incorrect. Nevertheless, we also analyzed NfL and GFAP levels in this distinct cohort and performed correlation analyses accordingly. The severity of nirAEs was graded for each patient from 1 (mild) to 5 (death caused by the adverse event), according to the Common Criteria for Adverse Events (CTCAE) version 5.0. Outcome of the (n)irAE was assessed in three categories as follows: (1) completely resolved symptoms, (2) resolved symptoms with sequelae or (3) persistent disability.

### 2.2 Serum analyses

Serum samples were collected from all patients during (n)irAE (timepoint: AE). Furthermore, for some patients, additional samples prior to (pre‐AE), during (AE) and after (post‐AE) the adverse event following ICI treatment were available for longitudinal analyses. Serum NfL and GFAP levels were determined using a single‐molecule array assay (Simoa® technology) as previously described.^18^ Serum NfL and GFAP levels were measured in all obtained patient samples. In total, 82 samples were measured in duplicate: 22 samples pre‐AE, 39 samples during AE, 13 samples post‐AE and eight samples during ICI therapy of cases with no irAE. For the nirAE groups, we were able to collect two samples pre‐AE, 19 samples during AE and one sample post‐AE. For irHypophysitis, 12 samples pre‐AE, seven samples during AE and nine samples post‐AE were available. For the control group with other irAEs, we collected eight samples pre‐AE, 13 samples during AE and three samples post‐AE. For the control group with no irAE, we collected eight samples during ICI therapy.

### 2.3 Statistics

Median and interquartile range (IQR) values were used to report the results. Continuous variables underwent assessment for normal distribution and equal variance via the D'Agostino–Pearson test. Between‐group comparisons were conducted for nonparametric data using the Mann–Whitney *U* test for two groups and the Kruskal–Wallis test with post‐hoc Dunn's testing for multiple groups. NirAE subgroup analysis to differentiate between CNSirAE, PNirAE and NMirAEs were based on control‐adjusted *Z* scores. The relationship between clinical parameters (age, CTCAE°) was evaluated using the Spearman's rank correlation coefficient (*r*), and correlational analysis was performed using simple linear regression (for the subgroups of nirAE and irHypophysitis). Statistical significance was determined at a level of *p* ≤ .05. Statistical analyses were executed with Prism (Version 9.5.1, GraphPad Software Inc., San Diego, CA) and R (2023.06.1 + 524) Figures were generated using Prism and Biorender (WX27EK597Q).

## 3 RESULTS

### 3.1 Patient population

In total, 53 cancer patients (age: 33–86 years; 35 male and 18 female) who received immunotherapy with ICI were included, 20 with nirAEs, 11 with irHypophysitis, 14 with non‐neurologic irAEs, and eight patients without irAEs (Table 1; some developed more than one (n)irAE). In patients with nirAEs undergoing ICI therapy, four demonstrated NMirAEs, five CNSirAEs and 15 developed PNirAEs.

**TABLE 1:** Patient characteristics.

Patient characteristics	nirAEs			irHypophysitis	Controls	
	Peripheral nirAE	Central nirAE	Neuromus‐cular nirAE		Other irAE	No irAE
*n*	11	5	4	11	14	8
Age med. (range)	57 (33–76)	77 (51–83)	72.5 (58–78)	70 (42–74)	75 (33–79)	76 (51–86)
Gender: female *n*–male *n*	3–8	4–1	1–3	4–7	5–9	1–7
*Tumor entity*						
Cut. melanoma	7	3	2	10	10	3
Uveal melanoma	1	0	0	1	3	0
NSCLC	0	0	1	0	0	2
SCLC	1	0	0	0	0	0
Urothelial c.	1	0	0	0	0	0
Renal cell c.	0	1	0	0	0	0
HCC	0	0	1	0	0	1
H‐Lymphoma	1	0	0	0	0	0
CRC	0	1	0	0	0	0
MCC	0	0	0	0	0	2
Mucosal mel.	0	0	0	0	1	0
*ICI*						
Pembro/Nivo	6	1	3	1	7	4
Ipilimumab	0	0	0	3	0	0
Ipi + Nivo/Pembr	4	3	0	7	6	0
oAtezolizumab	1	1	1	0	0	2
Avelumab	0	0	0	0	0	2
Tebentafusp	0	0	0	0	1	0
*CTCAE° irAE*						0
°1	0	0	0	0	0	
°2	2	0	0	4	10	
°3	8	1	2	3	3	
°4	0	4	1	0	0	
°5	0	0	1	0	0	
NA	1	0	0	4	1	
Multisystemic irAEs (*n* > 1)	10	3	1	10	9	0
*Survival* (*months*)		1 NA				
OS: 1st diagn.	26, 2 NA	30.5	51	116	43	37
OS: ICI start	22, 2 NA	12.5	36.5	33	24	34
PFS: ICI start	6, 2 NA	4.5	23, 1 NA	8, 3 NA	14	34, 1 NA
*Best response*						1 NA
PD	3	1	0	5	1	1
SD	3	1	1	3	10	3
PR	4	3	1	2	2	3
CR	1	0	2	1	1	0
Brain metastases	2	2	0	3	1	1

*Note*: Displayed are numerical values, with the median and range in brackets. The age at the beginning of treatment is presented. Median values for survival data are expressed in months. The best response is categorized according to Response Evaluation Criteria in Solid Tumors (RECIST 1.1).

Abbreviations: CRC, colorectal carcinoma; Cut. melanoma, cutaneous melanoma; HCC, hepatocellular carcinoma; ICI, immune‐checkpoint inhibitor; Ipi, Ipilimumab; NA, not available; Nivo, Nivolumab; (n)irAE, (neurologic) immune‐related adverse event; NSCLC, non‐small cell lung cancer; MCC, Merkel cell carcinoma; med., median; MUP, melanoma of unknown primary (MUP was included in cutaneous melanoma); OS, overall survival; Pembro, Pembrolizumab; PFS, progression free survival; SCLC, small cell lung cancer; TP, Timepoint of blood sample.

The most frequent of 11 underlying tumor entities in our cohorts were cutaneous melanoma (*n* = 35, 66%) and uveal melanoma (*n* = 5, 9%). Other tumor entities were diagnosed in 13 (24.5%) patients such as lung cancer (*n* = 4, 7.5%) or hepatocellular carcinoma (*n* = 2, 3.8%). In total, 22 (41.5%) patients received anti‐PD1 monotherapy including pembrolizumab or nivolumab, whereas 20 (37.7%) patients underwent a combined anti‐PD1 plus anti‐CTLA4 immunotherapy. Furthermore, 3 (5.7%) patients were treated with ipilimumab alone, 5 (9.4%) with atezolizumab, 2 (3.8%) with avelumab and one (1.9%) with tebentafusp. Details of patient characteristics are shown in Table 1.

Severe nirAEs (CTCAE° ≥3) were observed in 16 of 20 patients, including all five patients with CNSirAEs. Within the NMirAE cohort, one patient died related to the nirAE (CTCAE° 5), and for one patient with PNirAE, CTCAE grading was not available. In the control group, three out of 14 patients with other irAEs showed severe irAEs (CTCAE° ≥3). In the irHypophysitis cohort, three patients out of 11 developed severe irAEs (CTCAE° ≥3).

Overall, 33 (62%) patients developed irAEs with more than one organ affected. Therefore, a priorisation with respect to the clinically leading syndrome and most severe irAE has been established according to CTCAE grading. In the nirAE group, four out of 20 patients showed progressive disease (PD, RECIST 1.1), whereas 11 responded to ICI therapy including partial (PR) and complete responses (CR). Three patients of all had received a prior systemic tumor therapy, one patient with irHypophysitis and one patient of the other irAE group had received BRAF/MEK‐inhibitor therapy before, and one patient of the control group with other irAE had received tyrosine kinase inhibitor therapy, previously. In total, nine patients had developed brain metastases.

### 3.2 Serum NfL and GFAP levels in nirAE vs. other immune related subgroups

**FIGURE 2:**
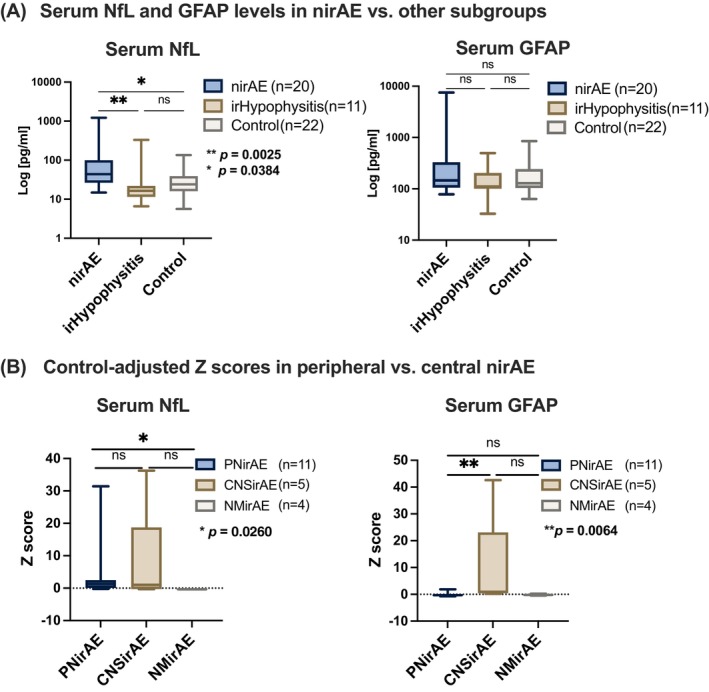
Serum NfL and GFAP levels in nirAE. (A) Serum NfL and GFAP levels in nirAE vs. other subgroups: Serum NfL levels were significantly higher in the nirAE group (*n* = 20) compared to irHypophysitis (*n* = 11, *p* = .0025, Kruskal–Wallis test with post‐hoc Dunn's testing) and other/no irAE control group (*n* = 22, *p* = .0384, Kruskal–Wallis test with post‐hoc Dunn's testing). GFAP levels did not significantly differ between groups, yet the nirAE subgroup exhibited a trend of higher GFAP levels. (B) Serum NfL and GFAP levels in different clinical manifestations of nirAE: Within nirAE subtypes (affected peripheral nerves/nerve roots: PNirAE, *n* = 11; central affection: CNSirAE, *n* = 5; or affection of the neuromuscular junction and/or muscles: NMirAE, *n* = 4) both PNirAE and CNSirAE showed increased NfL levels, while NMirAEs had lower NfL levels. Notably, PNirAE had significantly higher NfL levels than NMirAE (*p* = .0260, Kruskal–Wallis test with post‐hoc Dunn's testing). GFAP levels were significantly higher in CNSirAE compared to PNirAE (*p* = .0064, Kruskal–Wallis test with post‐hoc Dunn's testing). CNSirAE; affection of the central nervous system; GFAP: glial fibrillary acidic protein; NfL: neurofilament light chain; NMirAEs, affection of the neuromuscular junction and/or muscles; PNirAE; peripheral nerve/nerve root affection.

Notably, serum levels of NfL were found to be significantly higher in the nirAE group (*n* = 20, median 43.85 pg/ml, IQR: 26.45–99.4 pg/ml) compared to both the irHypophysitis (Figure 2A: *n* = 11; median 16.4 pg/ml, IQR: 11.5–21.9 pg/ml, *p* value: 0.0025, Kruskal–Wallis test and post‐hoc Dunn's test) and the control group with other or no irAE (Figure 2A: *n* = 22; median 24.08 pg/ml, IQR: 16.0–39.1 pg/ml, *p* value: 0.0384, Kruskal–Wallis test and post‐hoc Dunn's test). The serum levels of GFAP were not significantly elevated in the nirAE group compared to the other two groups. However, a further dissection of data revealed a tendency with higher GFAP levels in the nirAE cohort (Figure 2A: nirAE: *n* = 20, median 145.8 pg/ml, IQR: 105.15–329.95 pg/ml; irHypophysitis: *n* = 11, median 110.8 pg/ml, IQR: 100.3–204.0 pg/ml; control: *n* = 22, median 128.75 pg/ml, IQR: 107.3–240.38 pg/ml).

### 3.3 Serum NfL and GFAP levels in different clinical manifestations of nirAE

Within the heterogeneous nirAE group, classified by the clinical manifestations and phenotypes of the adverse event (i.e., CNSirAE: CNS‐affection; PNirAE: peripheral nerve/nerve root affection; NMirAE: affection of the neuromuscular junction and/or muscles), the following distinctive patterns emerged: Both PNirAE (*n* = 11, median: 75.3 pg/ml, IQR: 35.9–115.0 pg/ml) and CNSirAE (*n* = 5, median: 67.72 pg/ml, IQR: 26.1–359.3 pg/ml) displayed increased NfL levels. Interestingly, the NMirAE subgroup (*n* = 4, median 21.9 pg/ml, IQR: 15.45–27.0 pg/ml) exhibited significantly lower NfL levels (Figure 2B). Control‐adjusted *Z* score analyses between the subgroups revealed a significant increase in NfL levels in PNirAE compared to NMirAE (Figure 2B, *p* value: .0260, Kruskal–Wallis test and post‐hoc Dunn's test). However, no significant difference in NfL levels was noted between PNirAE and CNSirAE. Concerning GFAP levels, the highest concentrations were found in patients with CNSirAE (Figure 2B: *n* = 5, median 359.17 pg/ml, IQR: 281.54–2492.34 pg/ml), revealing a highly significant difference compared to those with PNirAE (Figure 2B: *n* = 22, median 143.1 pg/ml, IQR: 101.4–155.1 pg/ml) or NMirAE (*n* = 4, median = 164.3 pg/ml, IQR: 126.25–224.93, *p* value: .0064, Kruskal–Wallis test and post‐hoc Dunn's test).

### 3.4 Analysis of clinical parameters and serum NfL and GFAP levels

**FIGURE 3:**
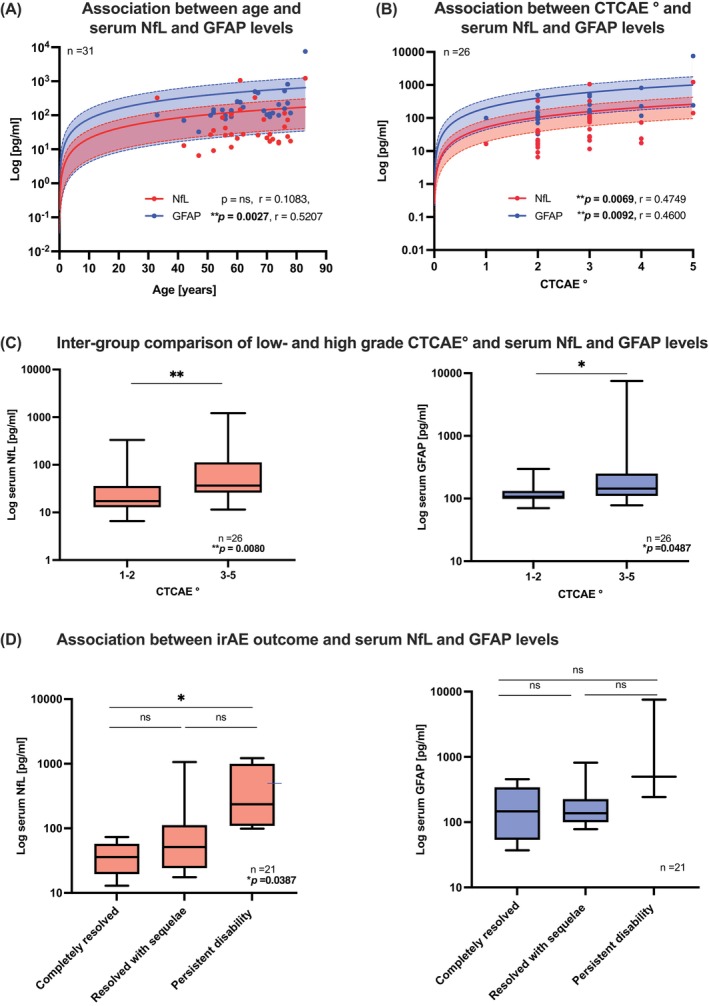
Analysis of clinical parameters and serum NfL and GFAP levels. (A) Correlation and linear regression analysis of age, and serum NfL and GFAP values: Age correlated significantly with GFAP levels in the subgroup of nirAE and irHypophysitis (*n* = 31, Spearman's *r*: 0.5207, *p* = .0027, confidence interval [CI] is given with 95% depicted with dotted lines and in between colored area). (B) Correlation and linear regression analysis of symptom severity and serum NfL and GFAP values: Symptom severity grading (CTCAE°) was available in 26 patients with nirAE or irHypophysitis and showed significant correlation with both NfL and GFAP serum concentrations (NfL: Spearman's *r* = 0.5207, *p* = .0069; GFAP: Spearman's *r* = 0.4600, *p* = .0092; for both regression analyses CI is given with 95%, depicted with dotted lines and in between colored area, red for NfL and blue for GFAP). (C) Group analysis between low‐grade (n)irAE (CTCAE° = 1–2) versus high‐grade (n)irAE (CTCAE° = 3–5) shows a significant difference for both NfL and GFAP levels (NfL: *p* = .0080, GFAP: *p* = .0487, Mann–Whitney *U* test). (D) Inter‐group analysis of prognostic implications of NfL and GFAP serum levels: Elevated NfL concentrations correlated with less favorable clinical outcomes in nirAE and irHypophysitis patients (outcome data was available in *n* = 21, categorized according to the following: Completely resolved symptoms, resolved with sequelae and persistent disability). Higher NfL levels were associated with less favorable outcomes: Group analysis between outcome parameters showed significant differences in their NfL levels between the status “completely resolved symptoms” versus “persistent disability” (*p* = .0387, Kruskal–Wallis test with post‐hoc Dunn's testing). There were no significant differences for GFAP levels. CI, confidence interval; CNSirAE, affection of the central nervous system; NfL, neurofilament light chain; GFAP, glial fibrillary acidic protein; NMirAEs, affection of the neuromuscular junction and/or muscles; PNirAE, peripheral nerve/nerve root affection.

Correlation and regression analysis were performed in the nirAE and irHypophysitis subgroups (in total *n* = 31) focusing on NfL and GFAP levels during the presence of the (n)irAEs. Spearman's *r* indicated a significant association between age and GFAP serum concentrations (Figure 3A: Spearman's *r* = 0.5207, *p* = .0027). In addition, symptom severity grading of nirAE, classified by CTCAE grading (available in *n* = 26 cases with nirAEs or irHypophysitis), correlated significantly with serum concentrations of both NfL and GFAP, as indicated by Spearman's r (Figure 3B: NfL: Spearman's *r* = 0.5207, *p* = .0069; GFAP: Spearman's *r* = 0.4600, *p* = .0092). Between‐group analysis regarding low‐grade (n)irAE (CTCAE° = 1–2) versus high‐grade (n)irAE (CTCAE° = 3–5) showed a significant difference for both NfL (NfL in low‐grade (n)irAE: median 17.20 pg/ml, IQR 12.90–35.90 pg/ml; NfL in high‐grade (n)irAE: median 36.60 pg/ml, IQR 26.08–112.3 pg/ml; *p* = .0080, Mann–Whitney U test) and GFAP levels (Figure 3C: GFAP in low‐grade (n)irAE: median 107.1 pg/ml, IQR: 98.75–132.7 pg/ml; GFAP in high‐grade (n)irAE: median 144.3 pg/ml, IQR: 110.1–250.2 pg/ml, *p* = 0.0487, Mann–Whitney *U* test).

To assess the potential prognostic implications of NfL and GFAP serum levels, we correlated them with outcome of (n)irAEs, which was classified into three categories: (1) completely resolved symptoms, (2) resolved symptoms with sequelae or (3) persistent disability. We could observe, that elevated NfL levels during the (n)irAE were associated with a less favorable outcome (Figure 3D). Furthermore, we found significant lower NfL levels in the group of patients with clinically “completely resolved symptoms” (Figure 3D: NfL median: 35.90 pg/ml, IQR: 19.60–57.65 pg/ml) compared to patients with “persistent disability” (Figure 3D: NfL median: 236.4 pg/ml, IQR 109.3–996.2 pg/ml; *p* = 0.0387, Kruskal–Wallis test with post‐hoc Dunn's testing). For GFAP levels we could not detect a significant difference, although we found higher GFAP levels in the group with “persistent disability” (Figure 3D).

### 3.5 Individual patient data and longitudinal analysis of NfL and GFAP concentrations in (n)irAE

Longitudinal analysis with at least two available timepoints was available for 19 patients, including nine with irHypophysitis, eight controls and two with nirAE. We noted a trend of slightly rising NfL levels correlating with the onset of nirAEs, which was not significant different compared to both the control group and irHypophysitis group (Figure S1). However, these observations are primarily descriptive, given the limited number of cases available for analysis.

## 4 DISCUSSION

NirAEs associated with ICI pose a significant challenge, potentially leading to permanent morbidity and mortality despite adequate side effect management. As ICI therapy extends to earlier tumor stages with increased long‐term survival rates, prompt identification and effective treatment of nirAEs becomes even more essential.^1^ In our multicenter study, we evaluated the diagnostic and prognostic utility of serum NfL and GFAP in patients with nirAEs.

We observed a significant elevation of serum NfL in patients with nirAEs compared to those with non‐neurological irAEs and no irAEs. Particularly, NfL levels were increased in nirAEs affecting the central nervous system (CNSirAEs) and peripheral nerves or nerve roots (PNirAEs), but not in those exclusively involving the neuromuscular junction and/or muscles (NMirAEs). However, GFAP, a marker of astrocyte activation, showed potential to differentiate CNSirAEs from other nirAEs not affecting CNS structures. Interestingly, elevated NfL levels in nirAEs and irHypophysitis correlated with the severity of neurological side effects and nirAE related outcome.

In our study, we focused on dissecting the combined potential of NfL and GFAP. While NfL is sensitive of neuroaxonal injury in general but lacks specificity regarding the site of neuroaxonal injury, GFAP specifically reflects astrocyte activation in CNS‐related pathologies. These findings are consistent with and extend those from prior reports:

NfL is a well‐established biomarker of neuroaxonal injury in various central and peripheral nervous system conditions.^29^, ^30^ Similarly, increased NfL levels have been noted in rare cases of nirAEs and neurotoxicity following CART‐cell therapy (ICANS).^18^, ^19^, ^20^, ^31^ Importantly, the severity of neurotoxic symptoms correlated with NfL levels, comparable to our findings.^18^, ^19^ Recent reports also found diagnostic utility of NfL, as it was shown to increase significantly during the occurrence of nirAEs, whereas monocyte chemoattractant protein 1 (MCP‐1) indicated an increased risk for nirAEs in the same study.^29^ Another study revealed that serum levels of NfL and S100B increased at the onset of nirAEs and returned to normal levels during immunosuppression.^32^, ^33^

In contrast to NfL, GFAP does not reflect neuroaxonal injury, but astrocyte activation evoked by CNS‐pathologies with primary or secondary inflammatory responses such as multiple sclerosis, traumatic brain injury, ischemic stroke, and Alzheimer's disease.^21^ Previously, increased serum GFAP levels were reported in single cases with nirAEs.^20^, ^34^ In addition, anti‐GFAP antibody mediated disease was observed in rare cases following ICI therapy.^35^, ^36^ Congruent to our initial hypothesis, a significant increase in GFAP levels was exclusively found in the subgroup with CNSirAE, but not in the other two subgroups with PNirAEs and NMirAEs. Thus, we could demonstrate the utility of GFAP in distinguishing nirAE subtypes, specifically CNSirAEs from other neurological side effects. Similar findings have recently been observed for GFAP in ICANS.^18^, ^19^ The prognostic value of GFAP in nirAEs, assessed through a regression model analyzing the severity of neurotoxic side effects and short‐term neurological outcomes, was found to be inferior to NfL in our study. This is not unexpected, given that GFAP is a highly specific biomarker of astrocyte activation and CNS pathology was a rare finding, while NfL reflects neuroaxonal injury more broadly, independent of the lesion site. In terms of prognostic significance, our findings align with previous studies that have demonstrated great utility of the combined assessment of NfL and GFAP correlating with disease activity and disability in multiple sclerosis.^37^

The classification of irHypophysitis as either a nirAE or an endocrine irAE remains a subject of ongoing discussion in the literature. In our study, we treated irHypophysitis as a separate cohort to prevent potential biases in the analysis of the nirAE subgroup. As irHypophysitis can present with various nonspecific neurological symptoms, such as headache, insomnia, or visual impairment, and often involves enlargement of the pituitary gland, a mild involvement of the CNS was not unreasonable.^20^, ^21^, ^22^ However, our study revealed no significant differences in NfL and GFAP levels between the irHypophysitis group and the control group. Thus, the status of irHypophysitis remains unclarified.

As demonstrated in this study, collaborative initiatives are needed to establish and gather information about rare and severe conditions like nirAEs, which necessitates close collaboration between centers, such as through the Side Effect Registry Immuno‐Oncology (www.serio-registry.org).^35^, ^36^ The analysis of a large cohort of patients with irEncephalitis has shown sequelae in half of affected patients and mortality in 10%–12.8% of patients.^38^, ^39^ Therapy in patients with nirAE not responsive to high dose corticosteroids includes IVIGs, infliximab, plasmapheresis and tacrolimus with scarce evidence on best second‐line therapy.^5^, ^40^ Unfortunately, as of today no baseline risk prediction is available and no precautions are available in clinical practice. Prospective studies will show whether combination of checkpoint inhibitors with, for example, anti‐IL6 can reduce irAEs and which is the best secondline therapy.^41^, ^42^

Despite the insights gained from our study, several limitations should be acknowledged. The relatively small study size, although bolstered by a multi‐center study, may limit the generalizability of our findings. Other relevant limitations for diagnostic accuracy include possible confounders impacting serum NfL and GFAP levels, such as previous neurologic disease or the presence of brain metastasis. In our analysis, we did not assess their potential confounding impact or exclude patients with brain metastasis from the study cohort. In terms of NfL, elevated levels have been associated with advancing age, particularly among males.^34^, ^35^, ^36^ Therefore, reference ranges adjusted for age are presently in the developmental stage.^37^ Nevertheless, we did not observe a significant correlation between age and NfL levels in our cohort. Other metabolic variables could have been confounding, as there is a known association between reduced muscle function and higher NfL levels, as well as a correlation of NfL with renal function.^43^, ^44^ We further demonstrate a potential correlation between GFAP and age, which is exemplarily known for healthy participants as well as patients with Parkinson's disease and multiple sclerosis.^45^, ^46^, ^47^ GFAP is known to be quite stable in individuals over time, whereas it is also influenced by gender, and the dynamics of blood levels depend on the acuity and development of the underlying CNS‐pathology.^21^

Initial serum measurements using earlier generation assays suffered from limited sensitivity. However, the latest immunoassay technology of Simoa, employed in this study, can accurately quantify NfL and GFAP even at low serum concentrations, with high reliability.^18^, ^48^ Thus, it could be easily implemented into prospective trials or even clinical practice.

## 5 CONCLUSION

NirAEs represent a clinically rare yet highly significant complication of ICI therapy. Timely recognition and treatment of nirAEs are crucial to avoid long‐term disability or even death. NfL and GFAP emerge as candidate biomarkers for the detection and subdifferentiation of the broad spectrum of nirAEs. Particularly NfL shows promise as a prognostic biomarker, correlating with the severity and outcome of nirAEs, thus being a potential tool to monitor the course and guide therapy of nirAEs. Further validation through larger and prospective studies is needed to confirm their clinical utility and facilitate their incorporation into clinical practice.

## AUTHOR CONTRIBUTIONS

**Christina Schmitt:** Conceptualization; methodology; data curation; validation; visualization; investigation; formal analysis; writing – original draft; writing – review and editing. **Katharina J. Müller:** Conceptualization; investigation; writing – original draft; writing – review and editing; visualization; validation; methodology; formal analysis; data curation. **Steffen Tiedt:** Validation; investigation; data curation; writing – review and editing. **Nora Kramer:** Investigation; validation; writing – review and editing; data curation. **Isabel Manger:** Investigation; validation; writing – review and editing; data curation. **Samuel Knauss:** Validation; writing – review and editing; investigation; data curation. **Leonie Müller‐Jensen:** Validation; writing – review and editing; investigation; data curation. **Petra Huehnchen:** Validation; writing – review and editing; investigation; data curation. **Wolfgang Boehmerle:** Investigation; validation; writing – review and editing; data curation. **Florian Schöberl:** Investigation; validation; writing – review and editing; data curation. **Lucie Heinzerling:** Validation; investigation; writing – review and editing; data curation; supervision; resources; project administration; software; conceptualization; methodology; funding acquisition; writing – original draft; formal analysis; visualization. **Louisa von Baumgarten:** Writing – review and editing; writing – original draft; funding acquisition; investigation; conceptualization; methodology; validation; visualization; project administration; formal analysis; software; data curation; supervision; resources.

## FUNDING INFORMATION

Funding was approved by BMBF (Bundesministerium für Bildung und Forschung) (grant no. 01ZX1905E) for our project MelAutim and by Bristol Myers Squibb foundation for SERIO (Side Effect Registry Immuno‐Oncology). It was further funded by the Berlin Institute of Health SPARK program (Wolfgang Boehmerle, Petra Huehnchen, Samuel Knauss, and Leonie Müller‐Jensen). Louisa von Baumgarten acknowledges support from the Deutsche Forschungsgemeinschaft (SFB‐TRR 338/1 2021–452881907), the Bavarian Center for Cancer Research (BZKF) and from the Bruno‐and Helene Joester foundation.

## CONFLICT OF INTEREST STATEMENT

NK declares financial support for congress participation from Sun Pharma. FS received honoraria from Amylyx, Alnylam and Alexion for advisory boards. LH declares speakers and advisory board honoraria from: Agenus, Bristol‐Myers Squibb GmbH & Co. KGaA, Huyabio, Immunocore Ireland Ltd., IO Biotech, MSD Sharp & Dohme GmbH, Novartis Pharma GmbH, Pfizer, Pierre Fabre Pharma GmbH, Regeneron, Replimune, Therakos (UK) LTD, Sol–Gel Technologies. All remaining authors have declared no conflicts of interest.

## ETHICS STATEMENT

The study has been approved by the local ethics committee of the Ludwig‐Maximilians‐University Munich (LMU) (No. 20‐1122, No. 20‐646), Friedrich‐Alexander University Erlangen‐Nürnberg (UKER) (No. 195_20 B) and Charité Universitätsmedizin Berlin (EA1/099/17; EA4/219/21). All participants gave orally and written informed consent according to the guidelines for Good Clinical Practice. The study was conducted in accordance with the Declaration of Helsinki. All centers were acting according to their regulatory requirements.

## Supporting information


**FIGURE S1.** Longitudinal NfL and GFAP serum levels in nirAE.

## Data Availability

The data that support the findings of this study are available from the corresponding author upon reasonable request.
