# Supplementary material for: Increased serum NfL and GFAP levels indicate different subtypes of neurologic immune‐related adverse events during treatment with immune checkpoint inhibitors
Source: Int J Cancer. 2025 Jan 20;156(10):1961–71. doi: 10.1002/ijc.35328 (PMC11924309; doi:10.1002/ijc.35328)
Supplement: Supplementary file 1 — FIGURE S1. Longitudinal NfL and GFAP serum levels in nirAE. [file IJC-156-1961-s001.pdf]

# Increased serum NfL and GFAP levels indicate different subtypes of neurologic immune-related adverse events during treatment with immune checkpoint inhibitors

Christina Schmitt, Katharina J. Müller, Steffen Tiedt, Nora Kramer, Isabel Manger, Samuel Knauss, Leonie Müller-Jensen, Petra Huehnchen, Wolfgang Boehmerle, Florian Schöberl, Lucie Heinzerling, Louisa von Baumgarten

Supplementary Material

Supplementary Figure 1

A)

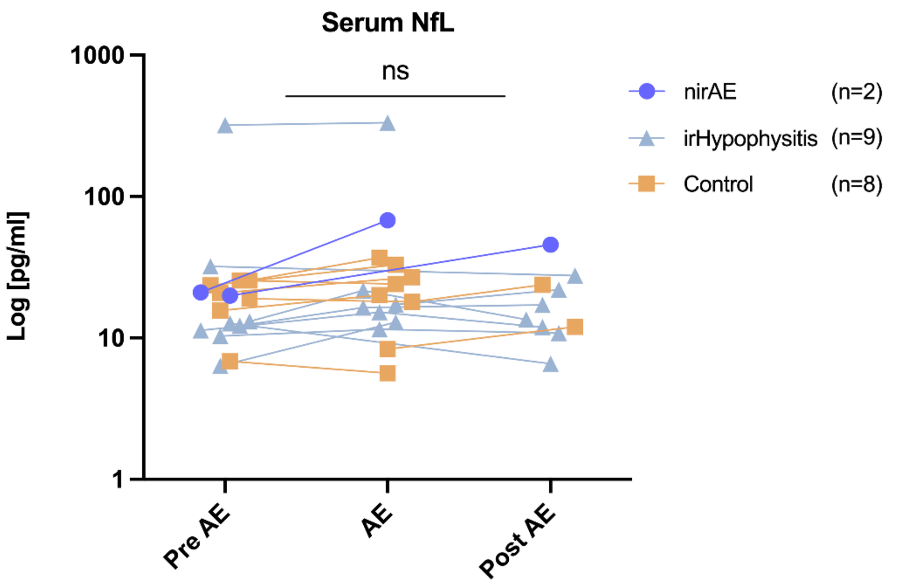


B)

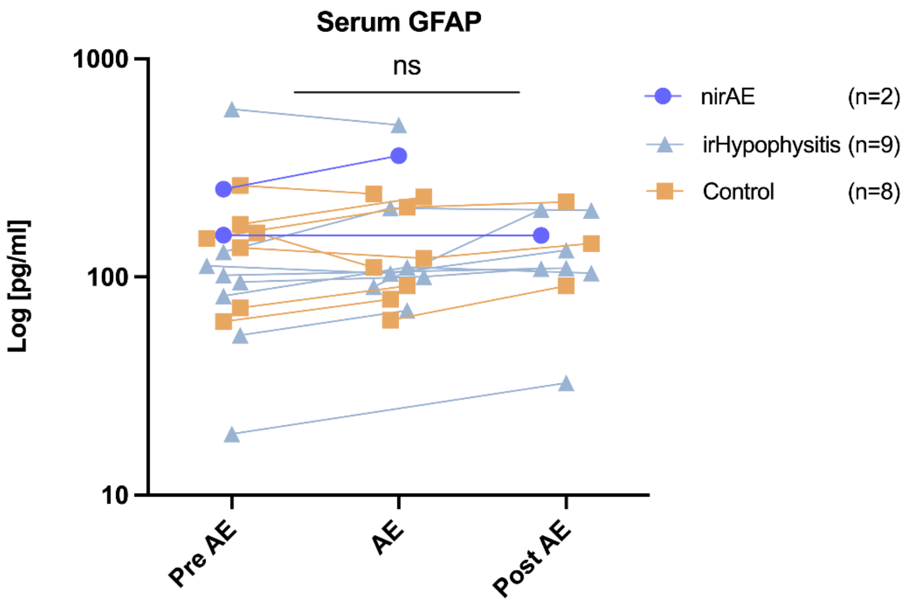


**Supp. Fig. 1 Longitudinal NfL and GFAP serum levels in nirAE**

Supp. Fig. 1 A), B) Longitudinal analysis of NfL and GFAP levels in nirAE: Limited longitudinal data (timepoints of analysis: pre-AE, AE, post-AE) showed a slight rise in NfL levels in nirAE (n=2), which was not significant different compared to controls (n=8) and irHypophysitis (n=9) cases.

*NfL: neurofilament light chain; GFAP: glial fibrillary acidic protein; AE: adverse event; nirAE: neurologic immune related adverse event.*
